# Breast Cancer Stem Cell Culture and Enrichment Using Poly(ε-Caprolactone) Scaffolds

**DOI:** 10.3390/molecules21040537

**Published:** 2016-04-23

**Authors:** Sònia Palomeras, Marc Rabionet, Inés Ferrer, Ariadna Sarrats, Maria Luisa Garcia-Romeu, Teresa Puig, Joaquim Ciurana

**Affiliations:** 1 New Therapeutic Targets Laboratory (TargetsLab)–Oncology Unit, Department of Medical Sciences, Faculty of Medicine, University of Girona, Girona 17071, Spain; sonia.palomeras@udg.edu (S.P.); m.rabionet@udg.edu (M.R.); ariadna.sarrats@udg.edu (A.S.); 2 Product, Process and Production Engineering Research Group (GREP), Department of Mechanical Engineering and Industrial Construction, University of Girona, Girona 17071, Spain; ines.iferrer@udg.edu (I.F.); mluisa.gromeu@udg.edu (M.L.G.-R.)

**Keywords:** breast cancer, cancer stem cell, scaffold, PCL, RepRap, tridimensional cell culture, mammospheres

## Abstract

The cancer stem cell (CSC) population displays self-renewal capabilities, resistance to conventional therapies, and a tendency to post-treatment recurrence. Increasing knowledge about CSCs’ phenotype and functions is needed to investigate new therapeutic strategies against the CSC population. Here, poly(ε-caprolactone) (PCL), a biocompatible polymer free of toxic dye, has been used to fabricate scaffolds, solid structures suitable for 3D cancer cell culture. It has been reported that scaffold cell culture enhances the CSCs population. A RepRap BCN3D+ printer and 3 mm PCL wire were used to fabricate circular scaffolds. PCL design and fabrication parameters were first determined and then optimized considering several measurable variables of the resulting scaffolds. MCF7 breast carcinoma cell line was used to assess scaffolds adequacy for 3D cell culture. To evaluate CSC enrichment, the Mammosphere Forming Index (MFI) was performed in 2D and 3D MCF7 cultures. Results showed that the 60° scaffolds were more suitable for 3D culture than the 45° and 90° ones. Moreover, 3D culture experiments, in adherent and non-adherent conditions, showed a significant increase in MFI compared to 2D cultures (control). Thus, 3D cell culture with PCL scaffolds could be useful to improve cancer cell culture and enrich the CSCs population.

## 1. Introduction

Breast cancer (BC) is the second most common cause of cancer-related death in women. Recent research has focused on a small population of the heterogeneous tumor cells that are responsible for tumor initiation and subsequent progression, the so-called Cancer Stem Cells (CSCs) [1]. Different studies revealed that these cells possess several characteristics similar to mammary stem cells [2], including radio- [3] and chemoresistance [4]. These properties favor breast cancer’s tumor recurrence. CSCs have the ability to undergo self-renewal and a potential to differentiate into non-stem breast cancer cells, generating cells with a variety of phenotypes within tumors [5]. The expression pattern of cells’ surface markers such as CD44 and CD24 has been used to isolate and enrich breast CSCs from the tumors [1,6]. Furthermore, these cells are able to grow and survive as non-adherent spheres, termed “mammospheres”, which enables their expansion in culture [7,8]. The study of CSC has been limited by the inability to propagate these cells without inducing differentiation, thus losing their stem-related features. The traditional two-dimensional (2D) cell culture systems are adequate to study cancer cells *in vitro*, but cannot completely simulate the *in vivo* cellular environment. This important difference in the cellular surroundings [9] may influence CSC properties and prevent their differentiation [10].

In recent years, three-dimensional (3D) cell culture has been developed to mimic the architecture of the extracellular matrix and the tissue environment, which can afford CSC culture without induction of differentiation. Scaffolds are one of the 3D culture systems, which are three-dimensional structures mostly made of polymeric materials. The use of biodegradable biopolymers as a structural 3D support material has emerged from using technologies already developed for this purpose, such as the Fused Filament Fabrication (FFF), widely used by 3D printers [11]. One of the most used biopolymers is poly(ε-caprolactone) (PCL), which exhibits suitable properties for tissue engineering, good mechanical characteristics, and relatively long-term biodegradability; it has also been proven to be biocompatible and free of toxic dyes [12]. Several studies have improved different mechanical, structural, and fabrication aspects of PCL tissue engineering scaffolds [13,14,15], but few studies have focused on cell attachment efficiency, proliferation, and differentiation within this 3D structure. Our previous studies investigated the optimization of the open-source and low-cost 3D extruder machine RepRap, employed to fabricate PCL scaffolds suitable for three-dimensional cell culture.

Cancer Stem Cells (CSCs) only represent a small population (10%–25%) of a tumor sample or cell line. CSCs are difficult to culture in 2D systems without inducing cell differentiation. To avoid this issue, this work focused on optimizing a three-dimensional culture protocol with a well-known breast cancer cell line (MCF7 cells). Scaffold cultures have been shown to provide a more physiological environment than monolayers (2D). Therefore, CSCs can grow with undifferentiated properties, while the rest of the sample cells remain differentiated. Consequently, a 3D culture can produce CSC enrichment compared to a 2D cell culture. Three-dimensional cell culture does not select or isolate CSCs, so an additional technique is necessary to quantify this population, the mammosphere-forming assay. Culture and medium conditions only allow the growth and proliferation of cells with CSC properties, forming spheres.

The present study has focused on the effect of different culture properties to improve scaffolds’ adequacy for breast cancer cells. In addition, the final objective of this work is to evaluate the CSCs’ enrichment due to scaffolds’ cell culture. Three-dimensional cell culture can be a useful way to enrich and isolate CSCs for further investigation targeted to this malignant subpopulation. According to this hypothesis, a higher cell proliferation could lead to a higher absolute number of CSCs.

## 2. Results

### 2.1. Scaffold Design and Manufacturing

In previous studies, design and manufacturing parameters were optimized to achieve high-quality scaffold printing, following a specific flowchart. All parameters have been modified considering the biopolymeric material characteristics and the printing process in order to optimize the porosity for cell culture.

Scaffolds were designed with a 19 mm diameter and a round shape to allow their use in regular cell culture plate dishes of 12 wells. Different design parameters were studied and analyzed, such as filament diameter, distance between filaments, and deposition angle (Table 1). The final designs had 2.4 mm of thickness, composed of eight different layers of polymeric material, each 0.3 mm thick.

The optimized fabrication parameters were extruder and bed temperature, deposition velocity, and layer height. These manufacturing parameters allowed accurate printing of the established designs.

The process parameters are shown in Table 1.

### 2.2. Scaffold Angle Design Evaluation

**Figure 1:**
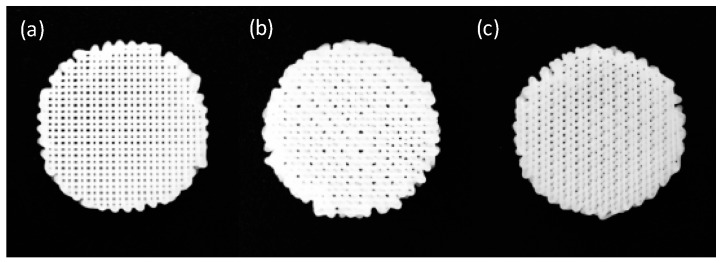
Three deposition angle designs fabricated with RepRap Machine. (**a**) 90° scaffold; (**b**) 45° scaffold; and (**c**) 60° scaffold.

Control size, geometry, interconnectivity, and spatial distribution of pores are critical parameters of scaffold designs [13]. Furthermore, pore parameters can influence the ability of cells to attach PCL filaments and fill the voids to create a 3D mass of cells. For these reasons, different pore designs were performed, selecting three deposition angles: 90°, 45°, and 60° (Figure 1).

**Figure 2:**
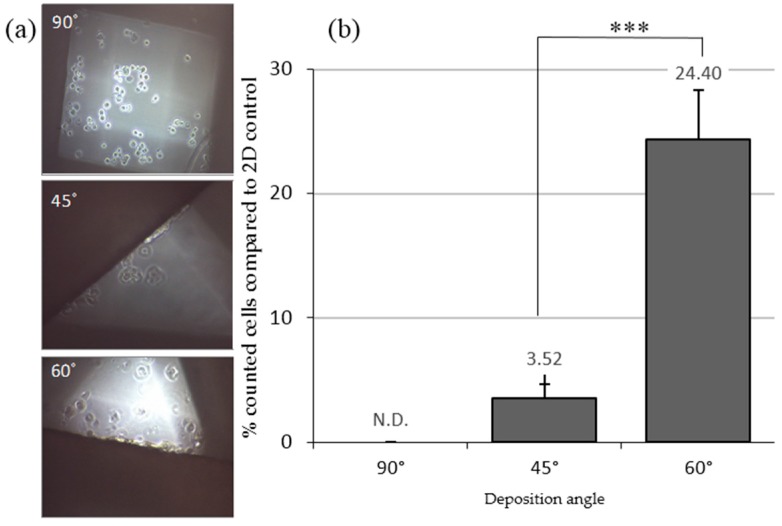
Cells cultured in 3D conditions with adherent well plates. (**a**) Inverted optical microscopy images of MCF7 cells seeded on different scaffold designs; (**b**) cells counted (%) three days after seeding in different deposition angle designed scaffolds. Results are shown as mean ± standard error. *** (*p* < 0.001) indicates levels of statistically significance.

MCF7 cells were seeded on the scaffolds and cultivated for 72 h in adherent culture plates. Cells were observed using an inverted optical microscopy (Figure 2a) and the attached ones were counted (Figure 2b). In 90° scaffolds, cells were only observed at the bottom of the well and non-attached cells were counted. Big pores may mean that cells can easily fall down to the bottom of the well before they can attach to PCL filaments. Attached cells were observed on the fibers of 45° and 60° designs, perhaps due to the smaller pore size. The cellular adhesion was higher in 60° (24.40% ± 1.16%) than in 45° (3.52% ± 3.96%) scaffolds.

The 45° and 60° scaffolds show adequacy for cell culture. In particular, the 60° design presented the highest percentage of cell attachment. For this reason, we utilized 45° and 60° scaffolds to study the ability of cells to attach to PCL filaments and discarded the 90° scaffold design.

### 2.3. Adherent and Non-Adherent Conditions for Scaffold Cell Adhesion

Nowadays, biocompatibility studies for *in vitro* assessment of scaffolds are very important. It is necessary to know the effects of tissue culture plate surface properties on cell adhesion to PCL filaments. To evaluate surface characteristics on seeding efficiency and cell growth in 3D culture, studies with adherent and non-adherent (pHEMA) wells were assayed. The same conditions were used in the 2D culture as in the control. There were no differences in cell morphology for those on scaffolds grown in both treated and non-treated well plates for the three days of the experiment.

**Figure 3:**
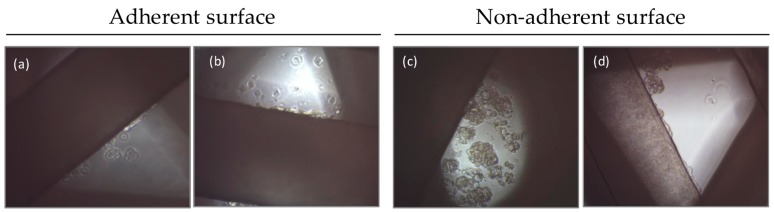
Inverted optical microscopy images of MCF7 cells seeded on scaffolds with adherent and non-adherent wells. (**a**) 45° and (**b**) 60° scaffolds in adherent conditions. Cells were attached both at scaffolds and at the surface; (**c**) 45° and (**d**) 60° scaffolds in non-adherent conditions. Cells were attached at the scaffolds and also formed suspension aggregates between the PCL filaments.

The attached cells were observed using an inverted optical microscopy. For both scaffold designs (45° and 60°), in adherent and non-adherent surface conditions, two types of cells were observed in the culture. In adherent conditions there were cells attached on the PCL filaments and cells attached at the bottom of the well (Figure 3a,b). Similarly, in non-adherent conditions cells were attached on PCL filaments and cells also formed suspension aggregates between the pores (Figure 3c,d). In both conditions cells were trypsinized and counted.

**Figure 4:**
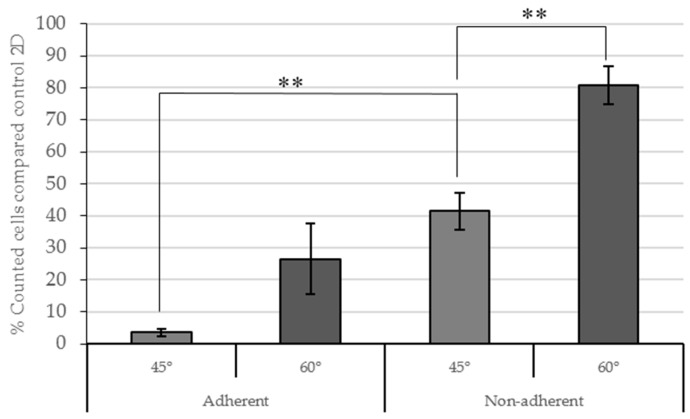
Counted cells (%) in 3D conditions compared to 2D control conditions three days after seeding. Adherent and non-adherent wells were assayed. Results are shown as mean ± standard error. ** (*p* < 0.01) indicates levels of statistically significance.

A higher percentage of counted cells with respect to the 2D control was seen in 60° scaffolds as compared to 45° scaffolds (Figure 4). In adherent conditions this difference was not significant (*p* = 0.28). In non-adherent conditions the percentage of cells in 60° scaffolds was significantly higher (*p* = 0.0006) in contrast to the 45° scaffolds.

Increased cell adhesion was observed on the scaffolds placed on a non-adherent surface compared to the scaffolds in adherent plates after 72 h seeding. The 60° scaffolds showed major cell adhesion in non-adherent conditions. However, the 45° scaffolds had significant differences (*p* = 0.006) between adherent and non-adherent conditions (Figure 4).

The number of cells attached at the bottom of the well in adherent conditions was then evaluated in both designs and compared to the 2D control. For 60° scaffolds, the percentage of cells was 9.04% ± 1.47% and for 45° scaffolds it was a little higher (11.78% ± 4.75%).

**Figure 5:**
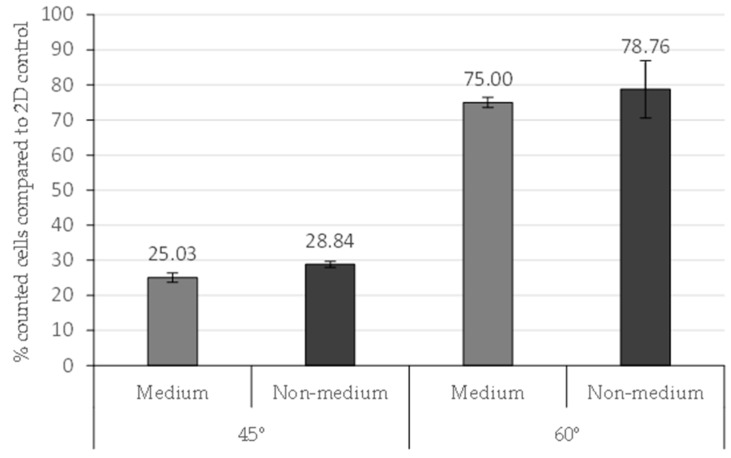
Counted cells in 45° and 60° scaffolds with previous medium or non-medium addition in the scaffold structure. Results are shown as mean ± standard error.

Based on the results, non-adherent conditions showed the highest cell attachment for both designs (45° and 60°). Some authors described a previous medium addition into the scaffold before cell seeding to facilitate cell attachment [15,16,17]. The medium addition was assayed to optimize cell adhesion in PCL filaments in non-adherent wells (Figure 5). Medium was placed on the center of scaffolds 30 min before cell seeding. A control without previous medium addition was also used.

This experiment enhances the previous results, showing an increased percentage of cells in 60° scaffolds compared to 45°. The medium addition did not modify the cell attachment in 45° and 60° scaffolds, with *p*-values of 0.082 and 0.691, respectively. In consequence, the previous medium addition was not established in the seeding scaffold protocol.

These results showed an optimal cell seeding efficiency and proliferation of 60° scaffolds.

### 2.4. Culturing MCF7 Cells on PCL Scaffolds Induces the Expansion of the CSC Subpopulation

Previous research reported that mammary epithelial stem and progenitor cells are able to survive and propagate in an attachment-independent manner and form floating spherical colonies, which are termed mammospheres [7]. Therefore, the number of spheres is often used to identify CSCs. To confirm whether the PCL fibrous scaffold culture system increases the CSCs population, we measured the Mammosphere Formation Index (MFI) of MCF7 cells previously cultured in 2D and 3D cultures with adherent and non-adherent conditions. Based on the results obtained in 3D culture studies, the deposition angle was fixed at 60°, because a 60° angle was the featured scaffold design with the highest proliferation rate.

Previous to the present study, an experimental procedure was performed to characterize the CSC population in the MCF7 line. It has been verified that the cell line of study was capable of forming mammospheres, with an MFI of 2.24% ± 0.23%.

**Figure 6:**
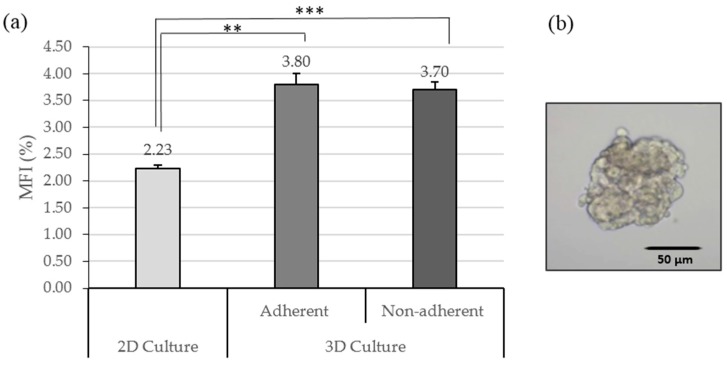
CSCs population analysis. (**a**) Mammospheres Forming Index (MFI; %) of 2D culture and all 3D conditions. Results are shown as mean ± standard error. (**b**) Inverted optical microscopy images of a MCF7 mammosphere. ** (*p* < 0.01) and *** (*p* < 0.001) indicate levels of statistically significance.

We found that the MCF7 cells from PCL fibrous scaffolds have increased the MFI compared with the control cells from a polystyrene surface (*p* = 0.003 for adherent conditions and *p* = 0.001 for non-adherent; Figure 6). There was no evidence of statistically different MFI in adherent and non-adherent conditions. Those data suggest that CSCs expanded in this mammary cancer cell line when cultured in scaffolds and a higher MFI is not related to the chemical characteristics of the surface well plate.

### 2.5. Flow Cytometry Optimization Analysis

Al-Hajj *et al.* reported the first phenotypic description of breast CSCs based on a high expression of CD44 and absent or low expression of CD24 on the cell surface (CD44^+^/CD24^−^/low phenotype) [1]. To determine the CSCs’ population enrichment in the 3D scaffold’s culture, flow cytometry assay was performed using antibodies against CD44-FITC and CD24-PE surface markers and using 7-AAD to assess cell viability. To achieve this aim, the flow cytometry protocol was optimized in MCF7 with a 2D culture.

**Figure 7:**
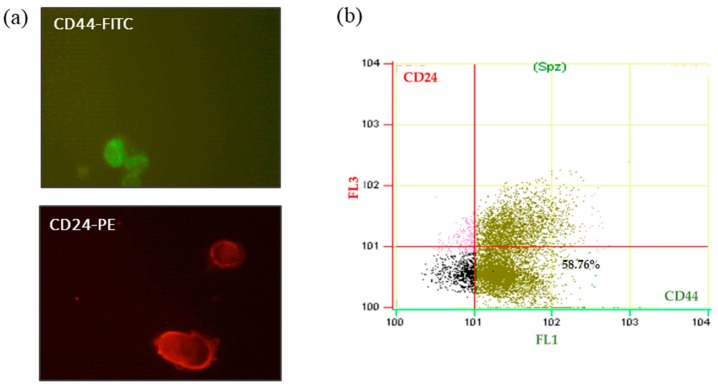
Expression of CD44 and CD24 cell surface markers on MCF7 2D cultured cells. (**a**) Confocal microscopy images of MCF7 cells marked with antibody CD24 and CD44 (Green: CD44-FITC; Red: CD24-PE); (**b**) flow cytometric analysis of CD24 and CD44 surface markers in MCF-7 cells from 2D culture system. Abbreviations: FITC, fluorescein isothiocyanate; PE, phycoerythrin.

To analyze the efficiency of antibodies against MCF7 cells, fluorochromes conjugated with the antibodies were tested. Cells were observed with a confocal microscope (Figure 7a). Positive cells for CD44 were green due to the emission spectrum peak wavelength of FITC (519 nm). CD24 positive cells, conjugated with PE, were red with an emission peak wavelength of 573 nm. In this preliminary analysis of MCF7, the purity of the CD44^+^/CD24^−^/low cell population in 2D culture was 58.76% (Figure 7b).

These preliminary results in 2D culture conditions might be useful in future studies characterizing the CSC subpopulation (CD44^+^/CD24^−^/low phenotype) generated in 3D scaffolds. Based on our literature review, we expect to observe an increase in CD44^+^/CD24^−^/low cells in breast cancer cells cultured in PCL scaffolds.

## 3. Discussion

CSCs have one of the most important roles in cancer and, for this reason, they must be specifically removed for a successful therapy. It has been reported that their frequency in primary tumors is correlated with the extent of tumor invasion and metastasis and, in turn, patients’ prognosis [1,2,18,19]. Therefore, their study has acquired great importance in recent years but it has been limited by the lack of physiologically relevant culture methods. Two-dimensional *in vitro* cell culture has been used in cancer research for many years. This monolayer culture can produce alterations in cell morphology and gene expression compared with those grown *in vivo* [10]. Tridimensional cell culture gives a more accurate morphological representation of tumor development and physiological environment. 

PCL scaffolds have been manufactured to test the cell adhesion efficiency of an MCF7 breast cancer cell line. The fabrication parameters used for PCL scaffold production show similar values to other studies. Domingos *et al.* set up a printing temperature of 80 °C, 10 mm/s velocity, and a layer height of 0.28 mm [20]. This combination of parameters generated meshes similar to the scaffolds used in this study. Furthermore, the effect of design parameter was evaluated through breast cancer cell culture in the current work. 

MCF7 culture in scaffolds showed the importance of the deposition angle in cell attachment and cell growth. The two scaffold designs with greater efficiency for cell culture, 45° and 60°, had smaller pore size and different pore shape compared with the 90° one. These results can be compared to those of Domingos *et al.*, who cultured a subtype of CSCs, the hMSCs (human Mesenchymal Stem Cells), in PCL scaffolds with different angle designs of 90°, 45°, and 60°. Their results showed more attached cells in 60° scaffolds then 45°, confirming the own results. However, they presented the 90° scaffold as optimal for cell culture with the highest values of cell adhesion. They showed that a higher deposition angle provides more space for cells to attach and proliferate [20]. The difference with the results obtained in the present assay may be due to the cell line studied. Specific cells require a different pore size for optimal attachment, growth, and motility [21]. Cell attachment is a complex process, affected by numerous aspects such as cell behavior, material surface properties, and environmental factors. 

In the current study, the efficiency of cell adhesion to the scaffolds has been evaluated based on the use of adherent and non-adherent wells. Polystyrene of the cell culture microplate was specifically treated to facilitate cell attachment. For non-adherent wells a commonly utilized polymer named poly(2-hydroxyethyl methacrylate) (pHEMA) has been used [22]. Once dried in the bottom of the well, it has neutral charge and is highly hydrophobic, avoiding cell attachment. Higher cell adhesion was observed on the scaffolds placed on non-adherent surfaces compared to the scaffolds on adherent plates. Blocking polystyrene treatment from adding pHEMA increased cell attachment in the PCL scaffolds. In consequence, cells only had the possibility of attaching to the PCL filaments of the scaffolds.

Tridimensional PCL fibrous scaffolds may offer an attractive alternative to culturing and propagating CSCs *in vitro*. In this study, it has been shown that tumor cells cultured in a 3D system displayed a significantly higher capacity to form mammospheres compared to the 2D control, revealing CSCs enrichment. The use of adherent and non-adherent wells did not affect the MFI. This variable has only altered the cells’ attachment to the scaffold rather than their ability to form mammospheres. Sims-Mourtada *et al.* and Feng *et al.* also showed a significant increase in the mammospheres formation index after scaffolds’ culture. In both works, the MFI after 3D culture was double the value obtained in 2D conditions. Sims-Mourtada *et al.* published that the MFI increase in a 3D culture was due to the differentiation inhibition of CSCs, since they did not observe an increase of the rate proliferation [23]. The second author suggested that CSCs’ enrichment was on account of the epithelial–mesenchymal transition (EMT), which can trigger the transformation of cancer cells to CSCs [15]. Others studies indicated that the malignant phenotype of cancer cells was also dramatically reduced when cells were transferred from *in vivo* conditions to 2D cell culture plates [24,25].

An important feature of breast cancer stem cells is the expression of the surface markers CD44 and CD24. Flow cytometry can be a powerful methodology to discriminate between populations and, therefore, determine the percentage of the CD44^+^/CD24^−^/low cells. The CSCs’ expansion due to tridimensional scaffold culture can be monitored in this way. At this point, staining and flow cytometry protocols for MCF7 cells have been developed.

Taken together, the presented results suggest that a 3D PCL scaffold culture spurred MCF7 cells to generate a cell population with CSC properties. Nevertheless, additional experiments are being developed with flow cytometry to determine the percentage of phenotype CD44^+^/CD24^−^/low as well as the analysis of the expression of genes related to epithelial–mesenchymal transition (EMT) expression. All of these assays are mandatory to characterize the population cells enriched in 3D PCL scaffolds, to study CSC properties, and to screen for new therapeutic agents targeting cancer stem cell populations.

## 4. Materials and Methods

### 4.1. Design and Manufacture of Scaffolds

#### 4.1.1. Filament

A 3 mm poly(ε-caprolactone) (PCL) wire (Perstorp, Malmö, Sweden) was used to manufacture the scaffolds. PCL has properties for tissue engineering with good mechanical characteristics, established biocompatibility, and relatively long-term biodegradability; it is also free of toxic dyes [12].

#### 4.1.2. 3D Printer Machine and Software

A three-dimensional RepRap BCN3D+ printer (Barcelona, Spain) was chosen to produce 3D scaffolds due to its open-source and modular related features. The machine has the capability to be modified and optimized by the user. This characteristic enable to print accurate geometric structures and reproducible scaffolds designs [11,26]. This printer used the Fused Filament Fabrication (FFF) technology, or so-called Fused Deposition Modeling (FDM), consisting of the deposition of the fused material in successive layers.

The designs of the scaffolds were performed with the computer-aided design (CAD) modeling software SolidWorks. The constructions were saved in STL file formats and transferred to Slic3r, computer-aided manufacturing (CAM) software, to establish the manufacturing parameters. Finally, this program generates G-code files capable of controlling the printer’s movements.

Design and manufacturing parameters were optimized for PCL and RepRap BCN3D+. Design parameters were established considering the scaffolds’ use in cell culture microplates with 12 wells (Table 2). Manufacturing parameters were determined in order to print the scaffolds efficiently and suitably for cell culture (Table 3). Both parameters were optimized by following a sequential flowchart.

#### 4.1.3. Deposition Angle Designs

**Figure 8:**
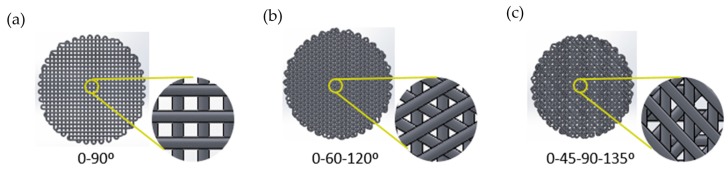
Scaffolds designs with different deposition angles: (**a**) 90°; (**b**) 60°; (**c**) 45°.

Three different scaffold designs have been performed with variable deposition angles, taking the values of 90°, 45°, and 60° (Figure 8). The variation of the angle of deposition between layers results in a different pore size and shape between the scaffolds (Table 4), which makes cell culture suitable or not.

### 4.2. Adherent and Non-Adherent Cultures

**Figure 9:**
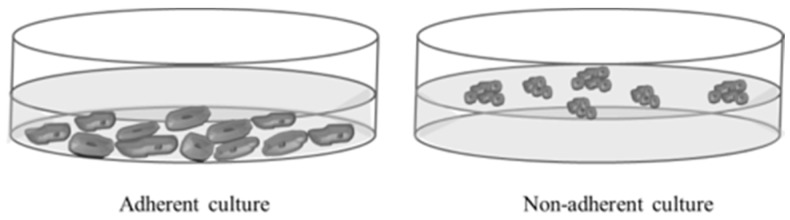
Schematic representation of adherent and non-adherent culture.

To study the effects of the well plate’s surface conditions (adherent *versus* non-adherent) on cell seeding efficiency and the subsequent enrichment of cancer stem cells in 3D PCL scaffolds, 12 multi-well surface plates were tested. The adherent surface conditions were tested in standard 12-well culture microplates. Non-adherent conditions were assayed with 12-well cell culture microplates treated with a hydrophobic and neutral charge polymer, poly(2-hydroxiethyl methacrilate) (pHEMA; Sigma Aldrich Co. LLC., St. Louis, MO, USA) (Figure 9). Non-adherent microplates covered with pHEMA were dried at 45 °C overnight and exposed to UV light for 20 min. The PCL scaffolds were divided in two groups and placed into the individual wells of adherent and non-adherent plates.

### 4.3. Cell Culture

**Figure 10:**
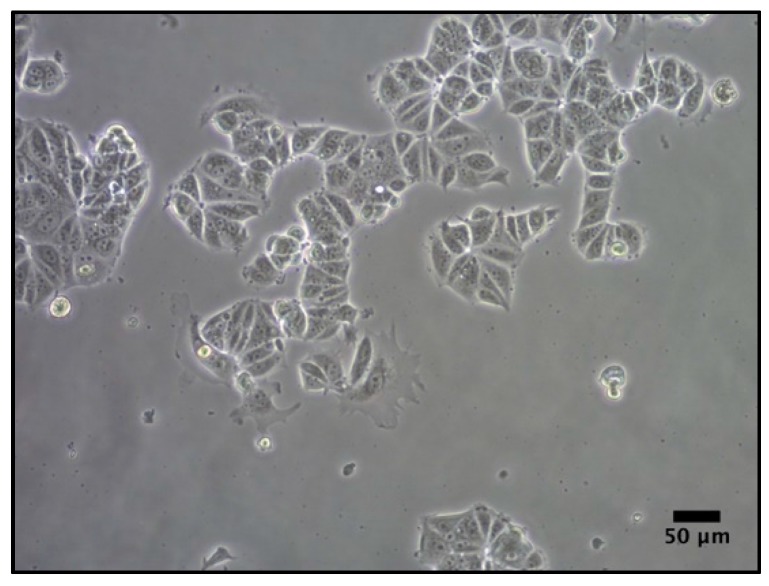
MCF7 cell line in 2D adherent culture.

MCF-7 breast carcinoma cells (Figure 10) were obtained from the American Type Culture Collection (ATCC, Rockville, MD, USA). MCF-7, is a HER2-positive cell line—a type of breast cancer that overexpresses Human Epidermal Growth Factor Receptor 2. Previous studies evaluated the biocompatibility of the scaffold with an MCF7 cell line [15,23].

Cells were cultured in Dulbecco’s Modified Eagle’s Medium (Gibco, Waltham, MA, USA) supplemented with 10% fetal bovine serum, 1% l-glutamine, 1% sodium pyruvate, 50 U/mL penicillin, and 50 µg/mL streptomycin (HyClone, Logan, UT, USA). Cells were maintained at 37 °C and 5% CO_2_ atmosphere.

Conventional cell culture was performed to study the cell proliferation in the different scaffold designs. For this reason, the medium was supplemented with nutrients and metabolites needed for the growth and proliferation of a vast majority of cancer cells, including MCF7.

### 4.4. Cell Culture in Scaffolds

#### 4.4.1. Scaffold Sterilization

Scaffolds were sterilized with 70% ethanol/water solution overnight, washed with PBS (Gibco, Waltham, MA, USA), and finally exposed to UV light for 90 min.

#### 4.4.2. Cell Culture in Scaffolds

**Figure 11:**
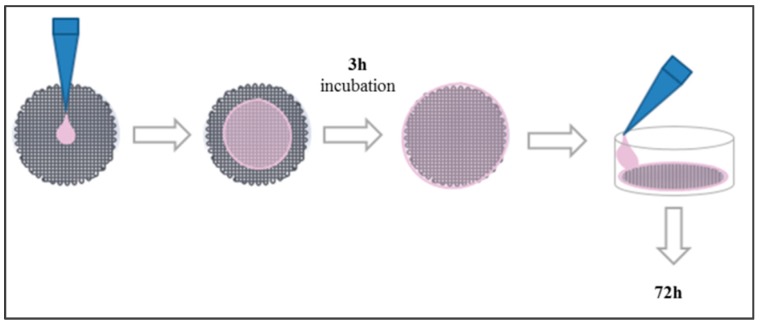
Cell seeding protocol on PCL scaffolds.

Scaffolds were placed into a 12-well cell culture microplate. First, 250 µL of cell suspension containing 100,000 cells were placed in the middle of its surface to allow cells to attach to the scaffold. After a 3 h incubation period, 1.5 mL of fresh medium were added to cover the scaffold (Figure 11). Cells were incubated for 72 h and then counted.

#### 4.4.3. Trypsinization and Cell Counting

The culture medium was removed with a micropipette. Then scaffolds were placed in new wells to quantify only the attached cells; they were washed with PBS and 1 mL of trypsin was added. After incubation, 2.5 mL of fresh medium were added and cell suspension was collected and centrifuged at 1500 rpm for 5 min. Finally, cells from the pellet were counted. The same procedure was done to obtain the number of cells attached at the bottom of the well. Cells were counted using a Neubauer Chamber (Marienfeld-Superior, Lauda-Königshofen, Germany) and an inverted optical microscope. Trypan blue solution was used to assess cell viability. This test measured the amount of viable cells, based on the concept that viable cells have an intact membrane and trypan blue cannot be incorporated. Dead cells have an altered membrane and take up the dye.

### 4.5. Mammosphere-Forming Assay

**Figure 12:**
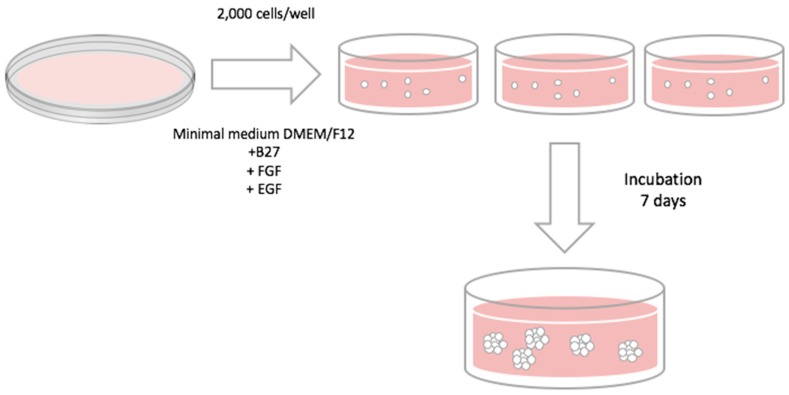
Mammosphere-forming assay protocol.

In order to evaluate CSC population, the mammosphere-forming technique was performed (Figure 12) as previously described [8]. Cells from 2D culture or 3D scaffolds were removed and re-suspended with DMEM/F12 medium supplemented with B27, EGF, and FGF (20 ng/mL), 1% l-glutamine, 1% sodium pyruvate, 25 U/mL penicillin, and 25 μM/mL streptomycin. Re-suspended cells were seeded into a six-well Cell Culture Microplate (Corning Life Sciences, New York, NY, USA) coated with pHEMA (Sigma Aldrich Co. LLC.) at a density of 2000 cells/well. Finally, cells were incubated for seven days and mammospheres bigger than 50 µm were counted using an inverted optical microscopy. The Mammosphere Forming Index (MFI) was calculated using the formula described below:

(1) $$MFI=\frac{N{}^{\circ}\;mammospheres}{\;N{}^{\circ}\;seeded\;cells}\;\times \;100$$

It is necessary to evaluate a subpopulation (CSCs) of a heterogeneous cell sample. This subpopulation is composed of undifferentiated cells with self-renewal characteristics. They are related to tumor initiation and they are capable of growth in suspension. For that reason, we used different medium supplements such as B27 (contains critical factors for cell survival and growth, excluding the use of serum), EGF, and FGF (growth factors). These are the culture conditions appropriate for allowing the growth and self-renewal of the CSCs.

### 4.6. Flow Cytometry Analysis

MCF7 cell growth on the polystyrene dishes was trypsinized as described above. Cells were re-suspended at a density of 1 × 10^5^ cells/mL in 25 µL of phosphate-buffer saline containing 2% of fetal bovine serum and blocked with human blocking reagent (Milteny Biotec, Bergisch Gladbach, Germany) for 10 min at 4 °C. Cells were stained with phycoerythrin-conjugated CD44 and fluorescein isohiocyanate-conjugated CD24 (BD Pharmingen, Franklin Lakes, NJ, USA) for 30 min at 4 °C in dark. After washing, cells were re-resuspended in a phosphate buffer containing 2% FBS and stained with 7AAD (BD Pharmingen) for 10 min at 4 °C. Samples were analyzed on a fluorescence-activated cell sorting Cell Laboratory QuantaSCTM cytometer (Beckman Coulter, Fullerton, CA, USA). Compensation was performed with single-stained cells to decrease overlaps of fluorophore emission spectrums.

### 4.7. Statistical Analysis

All data are expressed as mean ± standard error (SE). Data were analyzed by Student *t* test. Statistical significant levels were *p* < 0.05.

## 5. Conclusions

In the present study, different scaffold designs and culture parameters were tested on a breast cancer MCF7 cell line. Design of 60° and non-adherent conditions showed the highest cell counting after treatment with trypsin. CSC population was enriched in a tridimensional cell culture with 60° PCL scaffolds compared to the 2D culture control, increasing their MFI. The development of the flow cytometry analysis provides the basis for further studies to investigate CSC properties, as well as to screen new therapeutic agents targeting cancer stem cell populations. Using 3D culture, the total cell number will increase significantly and so will the CSC subpopulation. Then, we will perform cellular and molecular experiments to characterize the CSC population in order to find new therapeutic strategies against stem cells.

PCL scaffolds built with 3D printing machines revealed good results for cell cultures. Optimization of scaffolds’ porosity has been the key to cell growth and enrichment. This study suggests new criteria for enhancing bioprinting machines based on layer deposition.

## Figures and Tables

**Table 1:** Optimal process parameters values used for PCL scaffold printing.

	Parameters	Optimal Values
**Design Parameters**	Filament diameter	0.3 mm
	Distance between filaments	0.7 mm
	Deposition angle	90°, 60° and 45°
**Fabrication Parameters**	Extruder temperature	85 °C
	Bed temperature	35 °C
	Deposition velocity	10 mm/s
	Layer height	0.3 mm

**Table 2:** Design parameters and the corresponding optimized values.

Design Parameters	Optimal Values
Diameter	19 mm
Shape	Round
Number of layers	8
Distance between filaments	0.7 mm
Gap distance	0.4 mm
Deposition angle	90°, 45°, 60°
Filament diameter	0.30 mm

**Table 3:** Manufacturing parameters and the corresponding optimized values.

Manufacturing Parameters	Optimal Values
Deposition velocity	10 mm/s
Layer height	0.30 mm
Extrusion temperature	85 °C
Bed temperature	35 °C

**Table 4:** Pore characteristics depending on the deposition angle.

Deposition Angles	Pores Shape	Area
90°	Square	0.15 mm^2^
45°	Six variable forms (triangles and irregular polygons)	1.98 × 10^−4^ to 0.13 mm^2^
60°	Equilateral triangle	0.1256 mm^2^

## Abbreviations

The following abbreviations are used in this manuscript:

2D: Two-dimensional
3D: Three-dimensional
7-AAD: 7-AminoActinoMycin D
B27: B27 supplement
BC: Breast Cancer
CAD: Computer-Aided Design
CAM: Computer-Aided Manufacturing
CD24: Cluster of Differentiation 24
CD44: Cluster of Differentiation 44
CSCs: Cancer Stem Cells
DMEM: Dulbecco’s Modified Eagle’s Medium
DMEM/F12: Dulbecco’s Modified Eagle’s Medium Nutrient Mixture F-12
EGF: Epidermal Growth Factor
EMT: Epithelial–Mesenchymal Transition
FDM: Fused Deposition Modeling
FFF: Fused Filament Fabrication
FGF: Fibroblast Growth Factor
FITC: Fluorescein IsoThioCyanate
HER2: Human Epidermal growth Factor Receptor 2
hMSCs: Human Mesenchymal Stem Cells
MFC7: Michigan Cancer Foundation-7
MFI: Mammosphere Forming Index
PBS: Phospate-Buffered Saline
PCL: Poly(ε-caprolactone)
PE: PhycoErytrhin
pHEMA: poly(2-HydroxyEthil Methacrilate)
